# 

**Published:** 1869-04

**Authors:** 


Vol. V. *March & April*, 1869. No. 5.

### THE IOWA MEDICAL JOURNAL,

EDITED BY

### J. C. HUGHES, M. D.,

*Professor of the Institutes, and Practice of Surgery and Surgical Clinics in the Medical Department Iowa State University; Surgeon-in-Charge of the College Infirmary and Eye and Ear Institute.*

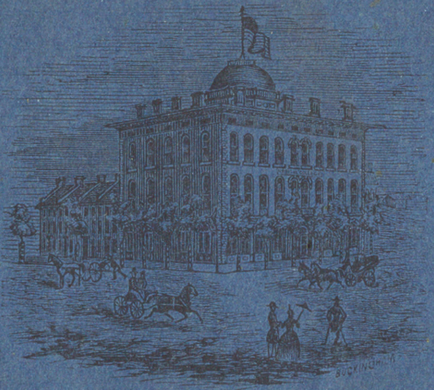

### KEOKUK, IOWA.

Editors and Publishers Please Exchange.

Address all Communications, Periodicals or Books to the Editor.

*KEOKUK, IOWA:*
PRINTED AT THE GATE CITY BOOK AND JOB ROOMS.
1869.

**Letters requiring an answer, must contain a Stamp to insure attention.**

